# Are GRU Cells More Specific and LSTM Cells More Sensitive in Motive Classification of Text?

**DOI:** 10.3389/frai.2020.00040

**Published:** 2020-06-30

**Authors:** Nicole Gruber, Alfred Jockisch

**Affiliations:** ^1^Department of Culture, Speech and Language, Universität Regensburg, Regensburg, Germany; ^2^Department of Information Technology, UKR Regensburg, Regensburg, Germany

**Keywords:** GRU, LSTM, RNN, text classification, implicit motive, thematic appeception test

## Abstract

In the Thematic Apperception Test, a picture story exercise (TAT/PSE; Heckhausen, 1963), it is assumed that unconscious motives can be detected in the text someone is telling about pictures shown in the test. Therefore, this text is classified by trained experts regarding evaluation rules. We tried to automate this coding and used a recurrent neuronal network (RNN) because of the sequential input data. There are two different cell types to improve recurrent neural networks regarding long-term dependencies in sequential input data: long-short-term-memory cells (LSTMs) and gated-recurrent units (GRUs). Some results indicate that GRUs can outperform LSTMs; others show the opposite. So the question remains when to use GRU or LSTM cells. The results show (*N* = 18000 data, 10-fold cross-validated) that the GRUs outperform LSTMs (accuracy = .85 vs. .82) for overall motive coding. Further analysis showed that GRUs have higher specificity (true negative rate) and learn better less prevalent content. LSTMs have higher sensitivity (true positive rate) and learn better high prevalent content. A closer look at a picture x category matrix reveals that LSTMs outperform GRUs only where deep context understanding is important. As these both techniques do not clearly present a major advantage over one another in the domain investigated here, an interesting topic for future work is to develop a method that combines their strengths.

## Introduction

The achievement Thematic Apperception Test, a picture story exercise (TAT/PSE; Heckhausen, 1963), is a very valid instrument for assessing the two components of the implicit achievement motive: hope of success (HS) and fear of failure (FF) (Schüler et al., 2015). In the test, people are instructed to invent stories based on six different pictures. The test person should do this by answering the following four questions: (1) who the persons in the picture are, (2) what they think and feel, (3) what happened before, and (4) how everything will turn out. Afterward, a trained psychologist classifies the stories for each picture regarding the absence or presence of 11 achievement motive categories. These are the need for success or to avoid failure (NS/NF), the instrumental activities to get success or prevent failure (IS/IF), the expectations of success or failure (ES/EF), specific positive or negative affect (A+/A–), failure outcome (F), praise (P), or criticism (C) (Heckhausen, 1963; annotation according to the English language translation by Schultheiss, 2001). There are also two weighting categories—so-called themes—that were given, when the story is more about success (ST) or about failure (FT). ST is scored when there is only NS or ES in the text and no other failure categories but A- or EF. The failure theme is scored when there is no other HS category but IS and at least NF or F is scored.

For each of the six pictures, these 11 categories are scored; afterward, they are summed up together with the themes to hope of success (NS+ IS+ ES+ A+ + P+ ST) or fear of failure (NF + IF + EF + A- + F + C + FT). The individual HS and FF scores for these six pictures are summed up again to the person's total HS and FF score.

As it is very complex to evaluate those picture story exercises, the need for computer evaluation of this test is given since a long time (Stone et al., 1966; Seidenstücker and Seidenstücker, 1974; Schultheiss, 2013; Halusic, 2015). Therefore, automatizing the evaluation of this assessment instrument combines two benefits: first, having a task that contains real human language understanding and, second, providing practical use.

**Figure 1:**
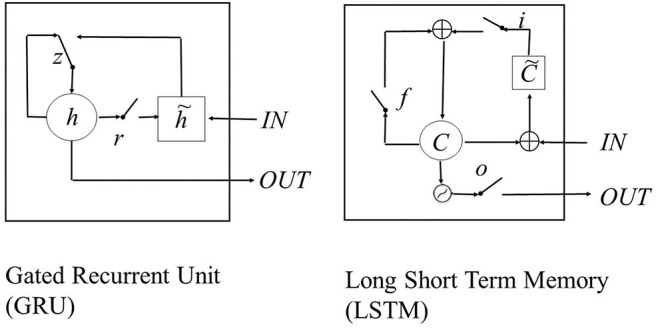
Gated recurrent unit (GRU; left) r = reset-, z = update gate, h = activation, $\tilde{\text{h}}$ = candidate activation, long short-term memory (LSTM; right) with input- (i), forget- (f), and output-gate (o), memory cell c, new memory cell $\tilde{\text{c}}$; (Schema cited after Rana et al., 2016, p 3; Chung et al., 2014, p 3).

Recurrent neuronal networks are commonly used for language understanding (Geron, 2018) as they are sequential data. There are different cells to improve neuronal networks when tracking long-term dependencies for deep sentence understanding. The first technique is the long-short-term-memory (LSTM) cell (Hochreiter and Schmidhuber, 1997). It has its own memory, which stores information outside the learning flow of the neural network. Thus, problems of long-term dependencies, i.e., in this case, for example, rarely frequented word combinations that are far apart, can be better controlled. The LSTM cell corresponds to a node of a recurrent network and has, in addition to the input and output, a forget gate that avoids overfeeding of the vanishing gradient. The second technique or alternative is the so-called gated recurrent unit (GRU; Cho et al., 2014); this function is performed via an update gate and a reset gate. The advantage of GRU cells is that they are just as powerful as LSTM cells (Chung et al., 2014) for moderately spaced word combinations, even with small data sets; but they need less computing power, so with the same equipment larger networks are feasible. With its three gates, the LSTM is more complex than the two-gated GRU cell. The LSTM has an input, output, and forget gate. The main difference is the presence or absence of an output gate, which tells how much of the content is presented to the next layer of the network. For LSTM cells, the whole memory can be limited by the output gate; in the GRU cell, this is not possible. In the GRU cell, this is handled via an update gate and a reset gate, where the update gate mostly does what in the LSTM is done by the input and forget gate. The reset gate handles the candidate activation in the cell. Therefore, in the GRU cell, the previous time step is more important. In the LSTM, there is no control of information flow in the cell as there is no reset gate. To put all in a nutshell, the GRU cell does not memorize as much as the LSTM cell, for it needs previous activation and remains in the network (see Figure 1). The LSTM cell could memorize more as it has one additional gate to control the output separately (Rana et al., 2016; Shen, 2017).

Some results indicate that GRUs can outperform LSTMs (Jozefowicz et al., 2015; Liu and Singh, 2016); some show opposite results (Amodei et al., 2015). So the question remains when to use GRU and when to use LSTM cells. We think that because of the architecture, the GRU cell is more sensitive for data and the LSTM is more specific. This perhaps could explain why in some cases GRUs outperform LSTMs and vice versa. So the aim of this study is to compare GRU and LSTM cells for automated motive coding, a special kind of text classification that requires deep text understanding, and look at sensitivity/specificity rates as well as accuracy rates depending on prevalence.

Based on the different architecture, we assume that

– The GRU cell outperforms the LSTM cell regarding accuracy in low prevalent content (<0.50).
– The GRU cell has higher specificity (true negative rate) than the LSTM cell.
– The LSTM cell should have higher sensitivity (true positive rate) than the GRU cell.

## Methods

### Data

As labeled data set for the study, 18000 stories (3000 tests, each one consisting of six picture stories A–F) were coded according to the 11 categories of the two components of the achievement motive hope for success (HS) and fear of failure (FF; Heckhausen, 1963). In part, they were received from other researchers and included archival data, where no information about gender and age is given. Therefore, also no ethical approval was done. The 18000 stories were coded toward the absence or presence of the 11 categories. As resulting labels, which are output by the neural network, we did not take the all-encompassing overall HS- or FF-scores but the coding regarding the 11 categories for each single picture story, because we wanted to get insights into the classification performance in different categories. We did not code each sentence separately as this does not comply with the guidance of Heckhausen (1963), and we wanted to consider the meaning of the text across the sentences. As we wanted to be sure that the coding of the data is consistent, seven students, who were also involved in data preparation, underwent 5 weeks of intensive training according to the guidelines of Heckhausen (1963). Their interrater agreement was calculated on a random data sample (*N* = 60), using an ICC (Shrout and Fleiss, 1979) with a two-way model on absolute agreement. It ranged from 0.70 to 0.73 (*p* < 0.001), which is an acceptable score (Meyer et al., 2002). The stories were sorted by person and picture for the analysis.

### The Network

For analysis, a recurrent neural network was created with an input and an output layer in tensorflow (https://www.tensorflow.org/, derived 06.04.2020). In the input, the text was inserted as a.txt file; the text was preprocessed by the natural language toolkit (www.ntlk.org, derived 06.04.2020).

For analysis, the words of the picture texts were encoded with the 64 dimensional word-embedding from polyglot, trained on German Wikipedia (https://sites.google.com/site/rmyeid/projects/polyglot, downloaded 06.04.2020). The concatenated word vectors were fed into a recurrent neural network, built with the tensorflow framework, consisting of an input layer, an inner layer, and an output layer.

For the comparison of the cell architectures, the vanilla RNN was replaced on the one hand by (1) the simple LSTM cell and on the other hand by (2) the GRU cell provided in tensorflow. The networks were trained in 1000 epochs without dropout, optimized by an Adam optimizer and a learning rate of 0.005; 1000 epochs were trained on the data with a 90% training set and a 10% testing set. The hyperparameters were chosen according to Geron (2018) and set by tensorflow. No individual tuning was done for both cell types to make sure that this would not influence the results at this stage. The procedure was as follows. The data were fed into the network. They were sorted by person (1–3000) and picture (1–6) as follows: Person 1, Picture 1, scoring category 1–11, Person 1, Picture 2, scoring category 1–11, Person 1, Picture 3, scoring category 1–11, Person 1, Picture 4, scoring category 1–11, Person 1, Picture 5, scoring category 1–11, Person 1, Picture 6, scoring category 1–11, Person 2, Picture 1, scoring category 1–11, …).

The picture text data were fed into the network (X-tensor); the scoring was set into the Y-tensor. It was sorted by person (1–3000) and then by picture (1–6). For every picture, the 11 categories were provided as the labels, which have to be learned. The training epochs were divided into mini-batches such that the data of a person were always together in the same mini-batch.

Training and testing sets were split according to this counter, so that in both sets, all six pictures were presented and no difference in content could influence the result. The classificatory was then trained to classify the text regarding the absence or presence of the 11 categories.

Afterward, a simple 10-fold cross-validation was performed. The resulting values were calculated per category and per picture as well as category x picture. The scores for testing the hypothesis that low prevalent content should be much easier to be learned by GRU and high prevalent content by LSTM, scored had to be transformed, because the original prevalence was below 0.50. Therefore, the sum-scores were dichotomized per picture or per category.

**Figure 2:**
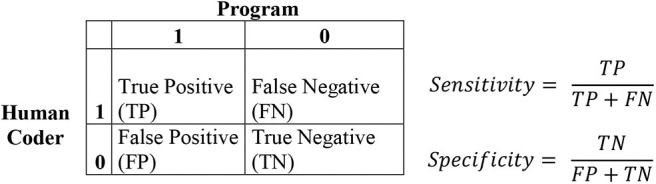
Classification method for the categories.

In addition to the accuracies, sensitivities and specifities were calculated (see Figure 2). As each category can be given a 1 (content is in category) or a 0 (content is not in category), there can be different decisions. If the program and the human coder decide that the specific content hits a specific category, this is named true positive on; the other hand, if both agree that it is not, it is true negative. If the program does not code a specific content, it is false negative. If the program codes the content and it would not be coded by the human, it is false positive. The sensitivity is calculated as the true positive rate of all decisions divided by all positive classifications, so true positive and false negative. The specificity is the true negative rate divided by all negative classifications, so false positive and true negative. So a high sensitivity concludes that the program is more likely to find, for example, a category that is scored. High specificity means that the program tends to not make a classification when there is nothing in the data. The prevalence was computed as the number of occurrences (cases coded as 1) divided by all cases. For example, if in 10 stories, there are five time-crossed category NS, the prevalence rate of NS would be 0.50. Prevalence is often indicated in %, but we worked with the decimals instead.

## Results

First the results show an overall testing accuracy of 0.85 for the GRU cell and 0.82 for the LSTM cell. But there were differences across the accuracy depending on the prevalence.

The results show that GRU cells tend to learn content that is rarely found in the data (low prevalence) better. LSTM cells, on the other hand, learn content that can be found more frequently in the data (high prevalence) better. As soon as the prevalence is balanced (~0.50), the classification performance adjusts (see Table 1).

**Table 1:** Comparison of GRUs vs. LSTMs regarding classification accuracy in a recurrent neural network based on 10-fold cross-validation for each picture (A–F; left) separated for the overall HS and FF score, as well as the HS (NS-A+) and FF categories (NF-F) depending on prevalence (Prev.) of the transformed scores.

	**Pictures (HS-score)**							**HS-categories**					
	**A**	**B**	**C**	**D**	**E**	**F**		**NS**	**IS**	**ES**	**P**	**A+**	
GRU	.606	.674	.545	.668	.515	.523	GRU	.487	.830	.716	.738	.492	
LSTM	.656	.649	.593	.626	.577	.562	LSTM	.525	.850	.687	.696	.540	
Prev	.722	.292	.652	.306	.685	.635	Prev	.525	.880	.282	.252	.538	
	**Pictures (FF-score)**							**FF-categories**					
	**A**	**B**	**C**	**D**	**E**	**F**		**NF**	**IF**	**EF**	**C**	**A–**	**F**
GRU	.756	.514	.617	.496	.617	.536	GRU	.598	.384	.545	.594	.513	.523
LSTM	.694	.534	.587	.560	.591	.554	LSTM	.591	.560	.547	.588	.554	.540
Prev	.241	.530	.389	.666	.371	.497	Prev	.394	.652	.461	.395	.581	.624

*The categories were need for success or to avoid failure (NS/NF), the instrumental activities to get success or prevent failure (IS/IF), the expectations of success or failure (ES/EF), specific positive or negative affect (A+/A–), failure outcome (F), praise (P), and criticism (C)*.

This is also found for categories. In all cases where the prevalence is >0.50, the LSTM cell outperformed the GRU cell; in all other cases, the GRU cell outperformed the LSTM cell with one exception: the EF category.

Table 2 shows that the specificity of the GRU cell is higher than the specificity of the LSTM cell. This can be found across all categories calculated for the pictures, regarding the HS-categories as well as the FF-categories. On the other hand, the sensitivity of GRU cells is always lower than the sensitivity of LSTM cells (see Table 2).

**Table 2:** Comparison of GRU vs. LSTM cells in classification sensitivity (true-positive-rate) and specificity (true-negative-rate) in a recurrent neural network based on 10-fold cross-validation (total sample 18000) for categories of Heckhausen (1963) regarding pictures (A–F; overall classification), HS-categories (NS−A+), and FF-categories (NF-F).

		**Pictures**						**HS-categories**					**FF-categories**					
		**A**	**B**	**C**	**D**	**E**	**F**	**NS**	**IS**	**ES**	**P**	**A+**	**NF**	**IF**	**EF**	**C**	**A–**	**F**
Specificity	GRU	.965	.965	.961	.951	.964	.952	.971	.721	.999	.997	.967	.992	.978	.990	.988	.946	.924
	LSTM	.937	.925	.921	.904	.924	.905	.913	.665	.984	.978	.915	.965	.858	.952	.966	.909	.909
Sensitivity	GRU	.340	.069	.172	.150	.198	.140	.036	.478	.001	.009	.063	.026	.027	.026	.034	.131	.212
	LSTM	.425	.153	.263	.223	.288	.215	.129	.557	.019	.060	.193	.083	.175	.101	.110	.214	.234

*The categories were need for success or to avoid failure (NS/NF), the instrumental activities to get success or prevent failure (IS/IF), the expectations of success or failure (ES/EF), specific positive or negative affect (A+/A–), failure outcome (F), praise (P), and criticism (C)*.

Similar results could also be found when separating for different categories (see Supplementary Table 1). An exception could be found when specific context information is needed to understand the coding. There are differences according to different categories and different pictures (Heckhausen, 1963).

For example, in picture B (a man in front of the director's office) in the category instrumental activity for success (IS) and negative affect (A–), the LSTM cell has higher scores for specificity than the GRU cell. This is because long-term dependencies are necessary to encode correctly. For example, if the person fears the director, this category will not be coded. If the person goes to the director with routine tasks, this category will not be coded.

Also the IS-category at picture D (a teacher and a student at a blackboard) is often coded false positive without background knowledge. The category must not be coded when the teacher writes something on the blackboard and corrects it, but only when the student does so.

Another category would be the category failure (F) in picture F (foreman and worker), because this category is only assigned if the one person who made the mistake cannot correct it himself or herself.

As a result, the superficial GRU cell codes this category less specifically but has a higher sensitivity than the GRU cell. Furthermore, the GRU cell has a higher sensitivity to the category negative affect (A–) in image C (two men at a workbench) than the LSTM cell.

The results show that in specific cases where complex additional information is needed to exclude the encoding, the LSTM cell encodes more specifically, and in situations where additional information from the text tends to confuse, the GRU cell cuts off more sensitively.

In pictures A (a smiling man at the desktop) and D (a man working at the desk), however, where only one person is depicted in the picture and only one person can be the protagonist of the story, the results are according to the assumptions across all categories.

## Discussion

In this paper, it is found that a potential automated motive coding by recurrent neural network technique could be possible. It is found according to the hypothesis that LSTM cells and GRU cells differ in the way they classify, and that this depends on the prevalence of the stimulus.

It is found that the performance of LSTM cells outperform GRU cells when the content to code is often represented in the data. This shows the difference in how these two memory cells work. Perhaps this could explain why in some cases GRU cells outperform LSTM cells and vice versa.

Furthermore, it is found that GRU cells should show higher specificity as they do not have their own memory and therefore tend to learn more like an exclusion principle. LSTM cells, on the other hand, show higher sensitivity as they strongly adopt onto the data. The only exception is found when the stories are complex (e.g., more than one protagonist of the story) and the context is important. This could lie on the fact that the LSTM, with its memory, could capture more information than the GRU.

As the LSTM cells are more likely to find the correct result categories, they are more sensible to overfitting than GRU cells. This could also explain why researchers found that GRU cells outperform LSTM cells when the sample size is low (Chung et al., 2014).

From a practical point of view, this short report enables new insights in memory cells in the way that the distribution of the target should be taken into account when choosing memory cells. So memory cells could be selected according to specific classification goals. For example, if GRU cells have higher specificities and lower sensitivities than LSTM cells, they would tend to overclassify rather than misclassify appropriate stimuli (alpha error), whereas LSTM cells would tend to overclassify rather than omit things (beta error). Thus, both types of cells offer specific practical advantages. This has to be tested in other contexts.

As a restriction, it has to be noted that this calculation is done only with less and specific data sets, so further research questions could be how the insights gained in this study about the difference of LSTM and GRU cells still work with larger data sets, more complex word embeddings, and more complex frameworks (e.g., deep learning). Further replications of this study will show how the GRU and LSTM cells also differ for other problems beyond implicit motive classification.

## Data Availability Statement

The datasets analyzed in this manuscript are not publicly available. Requests to access the datasets should be directed to nicole.gruber@ur.de.

## Author Contributions

NG wrote the first draft, ran the networks, and did the statistical analysis for this study. AJ refined the draft. Both authors contributed in optimizing the network architecture and approved the final manuscript.

## Conflict of Interest

The authors declare that the research was conducted in the absence of any commercial or financial relationships that could be construed as a potential conflict of interest.
