# Supplementary material for: Are GRU Cells More Specific and LSTM Cells More Sensitive in Motive Classification of Text?
Source: Front Artif Intell. 2020 Jun 30;3:40. doi: 10.3389/frai.2020.00040 (PMC7861254; doi:10.3389/frai.2020.00040)
Supplement: Supplementary file 1 [file Table_1.docx]

Supplementary Table 1

Comparison in classification sensitivity and specify of GRU vs. LSTM in a recurrent neural network for categories and pictures (A-F) and categories need for success of to avoid failure (NS/NF), the instrumental activities to get success or prevent failure (IS/IF), the expectations of success or failure (ES/EF), specific positive or negative affect (A+/A-), failure outcome (F), praise (P) or criticism (C)

|  | **Picture** | **Cell** | **HS-categories** | | | | | **FF-categories** | | | | | |
| --- | --- | --- | --- | --- | --- | --- | --- | --- | --- | --- | --- | --- | --- |
|  |  |  | NS | IS | ES | P | A+ | NF | IF | EF | C | A- | F |
| **Specifity** | A | GRU | .983 | .784 | .999 | .997 | .980 | .997 | .989 | .989 | .994 | .945 | .918 |
|  | A | LSTM | .895 | .671 | .981 | .983 | .909 | .958 | .853 | .916 | .951 | .911 | .891 |
|  | B | GRU | .969 | .632 | .999 | .996 | .959 | .983 | .973 | .983 | .990 | .877 | .939 |
|  | B | LSTM | .939 | .696 | .984 | .986 | .928 | .941 | .829 | .926 | .964 | .894 | .879 |
|  | C | GRU | .955 | .638 | .998 | .996 | .944 | .984 | .967 | .985 | .984 | .893 | .888 |
|  | C | LSTM | .863 | .563 | .969 | .974 | .883 | .915 | .757 | .874 | .925 | .856 | .822 |
|  | D | GRU | .964 | .664 | .999 | .995 | .962 | .991 | .972 | .994 | .993 | .946 | .921 |
|  | D | LSTM | .892 | .720 | .992 | .983 | .935 | .975 | .876 | .962 | .958 | .850 | .894 |
|  | E | GRU | .974 | .692 | .999 | .998 | .980 | .991 | .986 | .992 | .986 | .959 | .940 |
|  | E | LSTM | .909 | .664 | .987 | .983 | .919 | .962 | .865 | .969 | .975 | .906 | .930 |
|  | F | GRU | .966 | .780 | .998 | .995 | .978 | .991 | .987 | .976 | .990 | .971 | .919 |
|  | F | LSTM | .955 | .571 | .979 | .988 | .918 | .968 | .878 | .963 | .977 | .940 | .924 |
| **Sensitivity** | A | GRU | .019 | .463 | .001 | .010 | .043 | .014 | .023 | .019 | .026 | .192 | .272 |
|  | A | LSTM | .146 | .583 | .026 | .054 | .193 | .152 | .183 | .138 | .158 | .261 | .344 |
|  | B | GRU | .022 | .326 | .001 | .009 | .062 | .040 | .011 | .004 | .008 | .100 | .103 |
|  | B | LSTM | .042 | .360 | .010 | .023 | .083 | .064 | .113 | .067 | .076 | .144 | .160 |
|  | C | GRU | .042 | .356 | .001 | .001 | .045 | .012 | .017 | .009 | .042 | .055 | .124 |
|  | C | LSTM | .115 | .406 | .011 | .039 | .141 | .018 | .079 | .047 | .059 | .047 | .090 |
|  | D | GRU | .036 | .593 | .001 | .001 | .058 | .019 | .044 | .001 | .014 | .138 | .217 |
|  | D | LSTM | .112 | .484 | .016 | .045 | .139 | .057 | .169 | .096 | .114 | .281 | .231 |
|  | E | GRU | .024 | .527 | .001 | .005 | .062 | .005 | .018 | .012 | .022 | .082 | .133 |
|  | E | LSTM | .145 | .538 | .014 | .047 | .218 | .062 | .174 | .054 | .070 | .220 | .184 |
|  | F | GRU | .045 | .334 | .004 | .016 | .026 | .027 | .021 | .077 | .038 | .057 | .203 |
|  | F | LSTM | .087 | .641 | .017 | .078 | .186 | .066 | .142 | .097 | .047 | .128 | .173 |
